# NUDT15 genotyping during azathioprine treatment in patients with inflammatory bowel disease: implications for a dose-optimization strategy

**DOI:** 10.1093/gastro/goaa021

**Published:** 2020-06-26

**Authors:** Ye Xu, Yu-Qi Qiao, Han-Yang Li, Mi Zhou, Chen-Wen Cai, Jun Shen, Zhi-Hua Ran

**Affiliations:** Division of Gastroenterology and Hepatology, Key Laboratory of Gastroenterology and Hepatology, Ministry of Health, Inflammatory Bowel Disease Research Center, Shanghai Institute of Digestive Disease, Renji Hospital, School of Medicine, Shanghai Jiao Tong University, Shanghai, China

**Keywords:** NUDT15, azathioprine, inflammatory bowel disease, toxicity, efficacy

## Abstract

**Background:**

NUDT15 R139C is an Asian-prevalent genetic variant related to azathioprine (AZA) intolerance in patients with inflammatory bowel disease (IBD). However, it remains unclear how to utilize the genotyping results to improve the step-up dosing strategy with an already low starting dose in Asian practice.

**Methods:**

Clinical data of eligible IBD patients who received AZA therapy and NUDT15 R139C testing were retrospectively collected. The relationship between NUDT15 genotype, AZA doses, and AZA-induced toxicity and efficacy were comprehensively analysed.

**Results:**

A total of 159 patients were included for toxicity analysis. Compared with the wild genotype, patients heterozygous for R139C are more prone to developing myelotoxicity and alopecia (*P *=* *0.007; *P *=* *0.042). In particular, they had a 5.4-fold risk of developing myelotoxicity when AZA dosage was increased from 25 mg/d to 50 mg/d (*P *<* *0.001). Regarding efficacy, 115 patients who had received AZA for >4 months and maintained clinical remission on AZA monotherapy were included for further analysis. R139C heterozygotes were finally titrated to a significantly lower dose than the wild genotype [median (interquartile range): 0.83 (0.75–0.96) vs 1.04 (0.89–1.33) mg/kg/d, *P *=* *0.001], whereas the clinical remission rates did not differ between groups (*P *=* *0.88).

**Conclusions:**

IBD patients with R139C heterozygote are highly susceptible to AZA-induced myelotoxicity at an escalated dose of 50 mg/d. Thus, they may require a smaller dose increase after a starting dose of 25 mg/d. The final target dose of these patients could be set lower than that of the wild genotypes without compromising efficacy.

## Introduction

Azathioprine (AZA) is widely used as a first-line immunosuppressant in the treatment of inflammatory bowel disease (IBD) including ulcerative colitis (UC), Crohn’s disease (CD), and IBD-unclassified (IBD-U). This old drug has proven efficacy in steroid sparing and the maintenance of long-term remission [1, 2]. When additive to anti-TNF-antibodies, AZA has been found to exert an extra effect in suppressing the immunogenicity of biologics and thus accelerating mucosal healing. However, AZA has a relatively narrow therapeutic index and can result in substantial toxicity, particularly myelotoxicity, when overdose occurs [3].

The recommended standard dose of AZA in IBD patients is 1.5–2.5 mg/kg/d by European guidelines based on clinical trials featuring individuals of European descents [4, 5]. However, IBD patients in East Asia seem to have a poorer tolerance to AZA compared with Caucasians. Thus, rather than using a standard dose for all patients at initiation, most IBD physicians in East Asia start AZA at lower doses and then gradually titrate to a minimal effective dose to mitigate the risk of overdosing and toxicity [6–8]. Despite this, the incidence of AZA-induced leukopenia in East-Asian patients still reaches up to approximately 25% with an average low dose of AZA ranging from 0.8 to 1.1 mg/kg/d [9, 10], necessitating a search for better dosing methods.

To avoid overdosing, finding a marker to predict AZA-related toxicity would be beneficial. Thiopurine methyltransferase (TPMT) is a well-known enzyme that functions as an inactivator of the bioactive end products of AZA metabolism, 6-thioguanine nucleotides (6-TGN) including 6-thioguanosine 5'-triphosphate (6-TGTP), 6-thioguanosine 5'-diphosphate (6-TGDP), 6-thioguanosine 5'-monophosphate (6-TGMP) [11]. Genetic deficiency in TPMT is associated with AZA-induced leucopenia and increased levels of 6-TGN. Furthermore, pre-emptive genotyping of TPMT combined with 6-TGN monitoring has been successfully implemented to guide thiopurine dosing in European IBD patients, reducing the incidence of adverse events [12]. Nonetheless, the clinical usefulness of this strategy is rather limited in Asian IBD patients with a considerably lower frequency of TPMT mutation (∼1%) [13].

Recently, NUDT15 R139C has been identified as a crucial Asian-prevalent DNA variant that strongly predisposed patients to AZA-related leukopenia during IBD treatment, resulting in reduced dose tolerance [14, 15]. Yang *et al*. [14] showed that the incidence of early severe leukopenia in Korean patients homozygous for R139C was 100% at an initial dose of 25–50 mg/d during AZA treatment and thus AZA therapy is not recommended in mutant homozygotes of R139C. Nonetheless, a relatively wide variation of AZA tolerance has been observed in R139C heterozygotes [16]. Unfortunately, R139C is not associated with enhanced 6-TGN levels [3]. It remains vague how to adjust the dosage of AZA in IBD patients heterozygous for R139C in East Asia to minimize toxicity and maximize therapeutic efficacy.

Hence, we conducted this observational study to assess the relationship between NUDT15 genotypes and AZA-induced toxicity, especially AZA-induced myelotoxicity at different doses under a conventional step-up dosing strategy. In addition, we also examined the impact of NUDT15 genotypes on AZA final dose and efficacy, and thus aimed to provide a theoretical basis for NUDT15 genotype-guided individualization of AZA therapy from a combined perspective of both safety and efficacy.

## Patients and methods

### Patient selection

A total of 159 IBD patients admitted into Renji Hospital between August 2016 and January 2017 who had received NUDT15 and TPMT genotyping and AZA treatment were retrospectively identified. The inclusion criteria were: (i) diagnosis with CD, UC, or IBD-U; (ii) initiation of AZA treatment at our medical center; (iii) regular follow-up visits at our medical center. Exclusion criteria: (i) concurrent use of an immunosuppressant other than AZA; (ii) non-compliance in AZA administration. The study was approved by the Ethics Committee of Renji Hospital affiliated to Shanghai Jiao Tong University School of Medicine.

### AZA treatment

After excluding high-risk homozygotes (NUDT15 TT genotype or TPMT GG genotype), the initial oral dose of AZA was 25 mg/d. If patients were tolerable to 25 mg/d after 1–2 weeks, the AZA dose would be increased to 50 mg/d. After 3 months of 50 mg/d, the clinician would adjust the dose of AZA after a comprehensive evaluation of both tolerance and response. Thereafter, the medication plan was similarly adjusted according to both efficacy and toxicity every 3–6 months. If the patient continued to maintain remission, the evaluation interval could be extended as appropriate.

### Toxicity and efficacy evaluation

Myelosuppression was defined as white blood cell count (WBC) ≤3.5 × 10^9^/L or neutrophil absolute count (ANC) <2.0 × 10^9^/L. Myelotoxicity was defined as the occurrence of myelosuppression or acute decline of blood counts approximating the numerical criteria of myelosuppression that required drug discontinuation. The grading of myelosuppression was based on the Common Terminology Criteria for Adverse Events version 3.0 as follows: WBC <3.0 × 10^9^/L or ANC <1.5 × 10^9^/L for grade II and below; WBC <2.0 × 10^9^/L or ANC <1.0 × 10^9^/L for grade III and below; WBC <1.0 × 10^9^/L or ANC <0.5 × 10^9^/L was grade IV myelosuppression. Liver dysfunction was defined as alanine aminotransferase two times higher than the upper limit of normal or a short-term rapid rise required withdrawal of drug intervention. Pancreatitis was required to meet the diagnostic criteria for pancreatitis: the clinical symptoms combined with an increase in serum amylase of at least three times. After AZA initiation, a full blood count was performed once per week for the first month, once per 2 weeks for the second and third months, and then monthly thereafter. Routine assessments of liver and kidney function and serum amylase were taken every month.

Clinical remission was defined as Harvey–Bradshaw Index (HBI) ≤4 for CD and Simple Clinical Colitis Activity Index (SCCAI) ≤2 points for UC and IBD-U [17–19]. Duration of remission maintenance on AZA monotherapy was defined as consecutive months in clinical remission without concurrent use of steroids and infliximab (IFX).

### Data collection

Baseline patient information includes sex, ethnicity, type of IBD, genotyping results of NUDT15 and TPMT, age at diagnosis, disease location, history of intestinal resection, dates and doses of AZA at initiation, indication for AZA, type of induction therapy (enteral nutrition, steroids, IFX), concomitant or induction therapy [5-aminosalicylates (5-ASA)] at initiation, disease-activity indices (HBI for CD, SCCAI for UC and IBD-U) as well as follow-up data of AZA doses, blood tests, and disease-activity indices every 3 months were retrospectively collected via medical records. The peak dose of AZA was defined as the dose at which the patient developed an adverse reaction or the dose that was stable for 3 months or more at the last follow-up visits in non-encounters of adverse reactions. The final dose of AZA was defined as the dose that patients have used for 3 months or more at the last follow-up visits.

### Statistical analyses

The results in our study were analysed by the statistical package R version 3.4.3 (The R Foundation, Vienna, Austria) and Empowerstats (X&Y Solutions, Inc., Boston, MA, USA). Quantitative variables were expressed as median (interquartile range) and Mann–Whitney *U* test was used for comparison between groups. Categorical variables were expressed in terms of number and percentage and compared using chi-square or Fisher test. Ranked data were compared by Mann–Whitney *U* test. Hardy–Weinberg’s equilibrium was checked by chi-square test. Multivariate logistic regression was performed to control for potential confounding factors. Kaplan–Meier survival curves were plotted for estimation of the cumulative proportion of patients in sustained clinical remission and compared by the log-rank test. A *P*-value of <0.05 was considered statistically significant.

## Results

### Patient characteristics

A total of 164 IBD patients were initially identified. Three patients with incomplete clinical data and 2 who did not use AZA regularly as prescribed were subsequently excluded, leaving 159 IBD patients included in this study. The characteristics of the cohort are presented in Table 1 with all reporting Han ethnicity. The genotype frequencies of both NUDT15 and TPMT did not deviate from Hardy–Weinberg equilibrium.

**Table 1.** Characteristics of 159 patients with inflammatory bowel disease

Variable	Value
Age at AZA initiation, years, median (IQR)	32 (27–40)
Male gender, *n* (%)	113 (71.1)
Diagnosis, *n* (%)	
Crohn’s disease	131
Ulcerative colitis	24
Inflammatory bowel disease-unclassified	4
Peak dose of AZA, mg/d, median (IQR)	50 (50–75)
Peak dose of AZA, mg/kg/d, median (IQR)	1.00 (0.83–1.27)
Final dose of AZA, mg/d, median (IQR)	50 (50–75)
Final dose of AZA, mg/kg/d, median (IQR)	1.00 (0.83–1.17)
Duration of AZA use, months, median (IQR)	13 (6–22)
NUDT15 genotypes, *n* (%)	
CC	126
CT	33
TPMT genotypes, *n* (%)	
AA	155
AG	4

AZA, azathioprine; IQR, interquatile range.

### NUDT15 and adverse reactions

Details about adverse reactions and relevant clinical characteristics grouped by different NUDT15 genotypes are presented in Table 2. The peak doses (mg/kg/d) of patients with CT genotype were significantly lower than those with CC genotypes (*P* = 0.015). Except for peak doses, no other baseline characteristics significantly differed between the genotypes.

**Table 2.** Comparisons of adverse reactions and relevant factors between NUDT15 genotypes

Characteristic	CC genotype	CT genotype	*P*-value
	(*n* = 126)	(*n* = 33)	
Age at AZA initiation, years, median (IQR)	32.0 (28.0–40.0)	31.0 (25.0–44.0)	0.46
Male gender, *n* (%)	93 (73.8)	20 (60.6)	0.14
Diagnosis, *n* (%)			0.91
Crohn’s disease	103 (81.7)	28 (84.8)	
Ulcerative colitis	19 (15.1)	5 (15.2)	
Inflammatory bowel disease-unclassified	4 (3.2)	0 (0.0)	
Baseline WBC, ×10^9^/L, median (IQR)	6.12 (5.09–7.59)	7.16 (5.23–8.03)	0.37
Baseline ANC, ×10^9^/L, median (IQR)	4.09 (3.40–5.28)	4.84 (3.17–6.13)	0.38
Combined with corticosteroids, *n* (%)	79 (62.7)	21 (63.6)	0.92
Combined with infliximab, *n* (%)	13 (10.3)	4 (12.1)	0.76
Combined with 5-ASA, *n* (%)	45 (35.7)	12 (36.4)	0.95
Peak dose of AZA, mg/d, median (IQR)	50 (50–75)	50 (50–50)	0.001
Weight, kg, median (IQR)	58.0 (50.0–65.8)	55.0 (50.0–62.0)	0.26
Peak dose of AZA, mg/kg/d, median (IQR)	1.05 (0.84–1.35)	0.94 (0.79–1.11)	0.015
Observation time, months, median (IQR)	17.0 (13.9–20.1)	17.0 (14.6–19.4)	0.77
TPMT genotype, *n* (%)			1.00
AA	123 (97.6)	32 (97.0)	
AG	3 (2.4)	1 (3.0)	
Myelotoxicity, *n* (%)	28 (22.2)	15 (45.5)	0.007
Myelosuppression, *n* (%)	23 (18.3)	14 (42.4)	0.003
Grade I	13 (10.3)	7 (21.2)	
Grade II	8 (6.3)	5 (15.2)	
Grade III	2 (1.6)	1 (3.0)	
Grade IV	0 (0.0)	1 (3.0)	
Alopecia, *n* (%)	0 (0.0)	2 (6.1)	0.042
Infection, *n* (%)	4 (3.2)	2 (6.1)	0.605
Nausea and vomiting, *n* (%)	2 (1.6)	1 (3.0)	0.505
Liver dysfunction, *n* (%)	6 (4.8)	1 (3.0)	1.00
Pancreatitis, *n* (%)	2 (1.6)	0 (0.0)	1.00
Flu-like symptoms, *n* (%)	2 (1.6)	0 (0.0)	1.00

AZA, azathioprine; IQR, interquartile range; WBC, white blood cell; ANC, neutrophil absolute count; 5-ASA, 5-aminosalicylic acid.

The incidences of myelotoxicity and myelosuppression in CT genotype patients were significantly higher than that in CC genotype patients (*P* = 0.007 and 0.003, respectively). Besides, patients with CT genotype were more prone to alopecia than those with CC genotype (*P* = 0.042) and all occurred at a low dose of 25 mg/d. Other types of adverse reactions were not associated with the NUDT15 genotype.

### NUDT15 and myelotoxicity during dose adjustment

Information about NUDT15 genotype and myelotoxicity at different doses using a step-up dosing strategy is summarized in Table 3. There was no significant difference in myelotoxicity incidence between CT and CC genotype at an initial dose of 25 mg/d. However, when the dose was increased to 50 and 75 mg/d, the incidence of myelotoxicity in the CT genotype was significantly higher than CC genotype (*P* = 0.001 and 0.039, respectively). In the logistic-regression analysis with concomitant 5-ASA, weight, and CT genotype on the subgroup of patients whose doses were escalated to 50 mg/d, the NUDT15 heterozygotes had a 5.4-fold risk of myelotoxicity compared to wild-type patients (Table 4).

**Table 3.** Myelotoxicity and NUDT15 genotypes during dose adjustment

Dose of azathioprine	CC genotype		CT genotype		*P*-value
	No. of patients	Myelotoxicity, *n* (%)	No. of patients	Myelotoxicity, *n* (%)	
25 mg/d	126	2 (1.6)	33	0 (0.0)	1.00
33.3 mg/d	1	1 (100)	0	–	–
50 mg/d	121	16 (13.2)	30	12 (40.0)	0.001
75 mg/d	55	8 (14.5)	5	3 (60.0)	0.039
≥100 mg/d	20	1 (5.0)	0	–	–

**Table 4.** Logistic-regression analysis of risk factors for myelotoxicity at an AZA dose of 50 mg/d

Variable	Univariate analysis		Multivariate analysis	
	OR (95% CI)	*P*-value	OR (95% CI)	*P*-value
Age at AZA initiation (years)	1.0 (1.0, 1.1)	0.161	–	–
Female	1.2 (0.5, 3.0)	0.634	–	–
Combined with corticosteroids	0.7 (0.3, 1.7)	0.434	–	–
Combined with infliximab	1.0 (0.3, 3.8)	0.982	–	–
Combined with 5-ASA	2.5 (1.1, 5.7)	0.032	3.3 (1.3, 8.6)	0.012
Weight <50 kg	4.0 (1.6, 10.3)	0.004	5.7 (2.0, 16.6)	0.001
NUDT15 CT	4.4 (1.8, 10.8)	0.001	5.4 (2.0, 14.4)	<0.001

AZA, azathioprine; OR, odds ratio; CI, confidence interval; 5-ASA, 5-aminosalicylic acid.

### NUDT15 and efficacy

**Figure 1.**
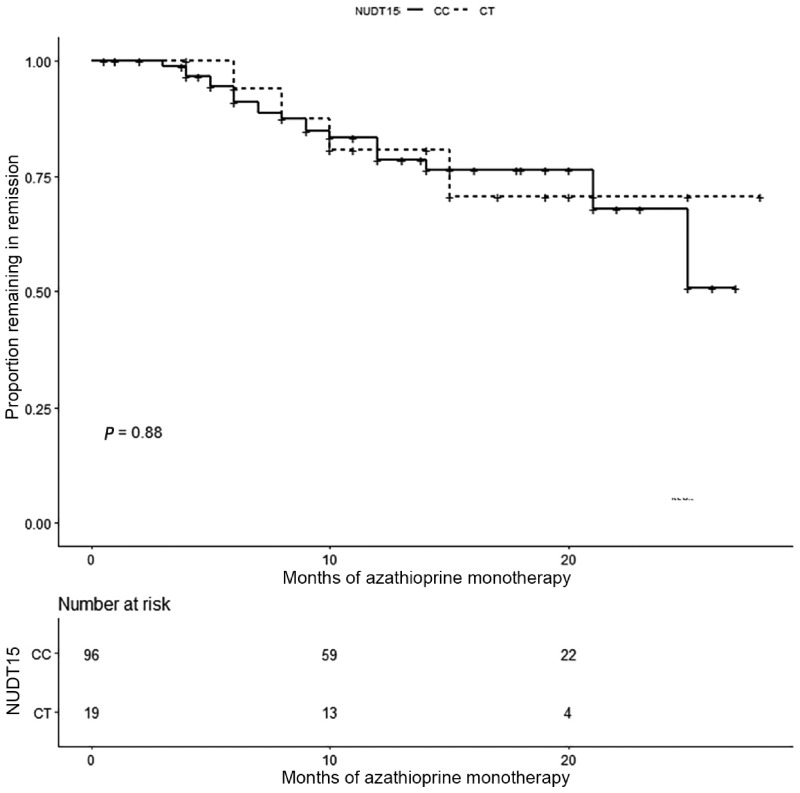
Comparison of cumulative remission rates between inflammatory bowel disease patients with NUDT15 CC genotype and those with CT genotype using Kaplan–Meier curves.

Only 115 who used AZA for 4 months or more and took AZA monotherapy for remission maintenance were further included for efficacy analysis. Information about AZA usage and relevant clinical characteristics between different genotypes is summarized in Table 5 and Supplementary Table 1 (according to the Montreal classification). Except that the final maintenance doses of patients with CT genotype were significantly lower than those with CC genotype, there was no significant difference in the baseline characteristics between the two groups. As for therapeutic efficacy, the proportion of patients with CC and CT genotype in clinical remission on AZA monotherapy was comparably the same in both genotype groups (78.4% vs 80.7% at 12 months, and 67.8% vs 70.6% at 24 months; log-rank *P *=* *0.88), as shown in Figure 1. Similar results were obtained in a subgroup analysis of patients with CD (75.3% vs 79.3% at 12 months, and 64.4% vs 68.0% at 24 months; log-rank *P *=* *0.78; Supplementary Figure 1). The sample of patients with UC and IBD-U was too small to analyse.

**Table 5.** Comparisons of clinical characteristics between different NUDT15 genotypes

Characteristic	CC genotype	CT genotype	*P*-value
	(*n* = 96)	(*n* = 19)	
Age at diagnosis, years, median (IQR)	28.0 (23.8–37.0)	26.0 (19.5–34.5)	0.39
Age at AZA initiation, years, median (IQR)	32.0 (26.8–39.0)	29.0 (25.0–35.5)	0.33
Duration from diagnosis to AZA treatment, years, median (IQR)	2.0 (0.0–5.0)	1.0 (0.0–4.5)	0.32
Male gender, *n* (%)	75 (78.1)	11 (57.9)	0.08
Duration of AZA use before monotherapy, years, median (IQR)	4.0 (0.0–4.0)	4.0 (2.5–4.0)	0.71
Diagnosis, *n* (%)			0.82
Crohn’s disease	81 (84.4)	18 (94.7)	
Ulcerative colitis	12 (12.5)	1 (5.3)	
Inflammatory bowel disease-unclassified	3 (3.1)	0 (0.0)	
Perianal disease, *n* (%)	42 (43.8)	9 (47.4)	0.77
Induction therapy, *n* (%)			0.70
Corticosteroids	64 (66.7)	14 (73.7)	
Infliximab	8 (8.3)	2 (10.5)	
Enteral nutrition	9 (9.4)	0 (0.0)	
Resection surgery	7 (7.3)	2 (10.5)	
5-ASA	4 (4.2)	0 (0.0)	
Others	4 (4.2)	1 (5.3)	
Final dose of AZA, mg/d, median (IQR)	75 (50–75)	50 (50–50)	<0.001
Weight, kg, median (IQR)	60.0 (52.0–68.0)	59.0 (52.5–62.5)	0.21
Final dose of AZA, mg/kg/d, median (IQR)	1.02 (0.88–1.25)	0.83 (0.76–0.95)	0.001
TPMT genotype, *n* (%)			1.00
AA	94 (97.9)	19 (100.0)	
AG	2 (2.1%)	0 (0.0)	

AZA, azathioprine; IQR, interquartile range; 5-ASA, 5-aminosalicylic acid.

## Discussion

NUDT15 R139C has recently been established an important pharmacogenetic predictor of AZA intolerance in East-Asian IBD patients [15, 20]. Clinical guidelines from the Clinical Pharmacogenetics Implementation Consortium suggest using a reduced starting dose in R139C carriers to prevent AZA-induced leukopenia [21]. However, it remains obscure to what extent a final dose reduction is required and whether this would impair the therapeutic efficacy during IBD treatment. In fact, most Asian practices have long used a low starting dose of AZA (25–50 mg/d) that was subsequently increased to a local target dose based on tolerance and efficacy [6–8], but the incidence of adverse reactions, especially myelotoxicity, remains high [9, 10]. In the present study, we attempted to provide new insight into AZA dose optimization on the basis of this conventional dose-escalation strategy and the application of NUDT15 genotyping. In toxicity analysis, we evaluated the appropriateness of the starting dose and rapidity of dose escalation in different R139C genotypes under the step-up dosing strategy and inferred that the usual dose increment of 25 mg/d is very likely to cause undesirable overdosing of AZA in R139C heterozygotes in terms of safety. Furthermore, our study provides the first direct piece of evidence supporting that the final target dose of these heterozygous patients could be set lower than that of the wild genotypes without compromising efficacy during IBD treatment.

Given that the incidence of severe early leukopenia is approximately 100% in R139C homozygotes [15], patients who were mutant homozygous for R139C were precluded from receiving AZA if they received pre-emptive genotyping in our medical center. Except for these patients, all the remaining patients started AZA at a considerably low dose of 25 mg/d and subsequently adjusted it according to both toxicity and efficacy, as a routine part of clinical practice. In this context, the incidence of myelotoxicity and alopecia, compared with wild-genotype patients (CC genotype), was still significantly higher in R139C heterozygotes (CT genotype) and the myelotoxicity rate reached as high as 45.5% in the heterozygous group despite the significantly lower doses. These data are well in line with the results of previous pharmacogenetic studies in East-Asian populations with variable starting doses [9, 10, 22], reaffirming the association between NUDT15 R139C and AZA-induced toxicity in Chinese IBD patients. On the other hand, our finding indicates that a step-wise dose adjustment with a low starting dose of 25 mg/d is insufficient to shrink the great disparity in the incidence of myelotoxicity between patients with CC and CT genotypes.

Are there any changes that could be made to modify this conventional dosing strategy to reduce AZA-induced myelotoxicity? To better address this question, we performed a stratified risk analysis of myelotoxicity according to dose adjustment in real clinical settings. At an initial dose of 25 mg/d, the incidence of myelotoxicity was extremely low in both genotypes. However, when the dose was directly increased to 50 mg/d and from 50 to 75 mg/d, patients with NUDT15 CT genotype had a significantly higher incidence of myelotoxicity than those with CC genotype (40% vs 13.2%, *P* = 0.001; 60% vs 14.5%, *P* = 0.039). The multivariate analysis of patients whose dosage was escalated from 25 to 50 mg/d revealed that patients with CT genotype has a 5.4-fold risk of myelotoxicity in comparison with the CC genotype. These results suggest that the starting dose of 25 mg/d is safe but further dose escalation to 50 mg/d may be too large a step in terms of toxicity in R139C heterozygotes using the routine dosing strategy. Thus, a smaller dose increment after 25 mg/d may be more appropriate for heterozygotes, such as from 25 to 33.3 mg/d (take two pills of AZA every 3 days with a unit dose of 50 mg/d) to prevent overdosing. If one heterozygous patient was receiving a NUDT15 genotyping test but was still directly titrated to a dose of 50 mg/d, pre-emptive genotyping might not be helpful in reducing the high myelotoxicity rate, whereas patients with CC genotype are relatively safe to be titrated to 50 mg/d if no sign of drug intolerance is detected at a starting dose of 25 mg/d.

Regarding efficacy, as NUDT15 heterozygosity is reportedly not associated with high 6-TGN levels, it is unclear how to optimize the target dose in the CT genotype due to a lack of other mature metabolite markers to predict potential efficacy [3]. In the present study, a low dose of AZA was prescribed in most IBD patients under the step-up dosing strategy and the final AZA maintenance dose was further reduced in patients with CT genotype compared with those with the wild genotype [0.83 (0.75–0.96) vs 1.04 (0.89–1.33) mg/kg/d, *P* < 0.001]. Notwithstanding, the cumulative proportions of patients in remission maintenance on AZA monotherapy were similar between patients with different genotypes. In the CD subgroup, the remission rates at 12 and 24 months in the two genotypes are comparable with previous reports that evaluated the clinical efficacy of a standard dose of AZA in remission maintenance [23–25]. These observations are in accordance with the long-standing notion that the gradual titration strategy and subsequently low doses of AZA are effective in East-Asian populations [8, 26]. More importantly, these results indicate that further dose reduction does not compromise the clinical efficacy of AZA in IBD patients with CT genotype in comparison with CC genotype.

Interestingly, a similar finding has been documented when dating back to earlier studies in individualization therapy in IBD patients based on TPMT genotyping. In an observational study, Gardiner *et al*. [27] first demonstrated that, when the doses were individually adjusted by 6-TGN, the final average dose in TPMT heterozygotes was only half that in the wild genotype (0.9 vs 1.8 mg/kg/d) without impaired efficacy. Recently, TOPIC trials have revealed that, within a 20-week observation time, TPMT heterozygotes can achieve effective 6-TGN levels with a final average dose of 1.0 mg/kg/d and have comparable remission rates with wild-genotype patients at an average dose of 2.2 mg/kg/d [12]. Although NUDT15 is different from TPMT, as it does not influence the total concentration of 6-TGN, it can convert 6-TGTP to 6-TGMP with its polyphosphate hydrolase activity [28]. 6-TGTP is regarded as the predominant component of 6-TGN associated with immunosuppressive function [3], which can specifically inhibit Rac1 activation, leading to T-cell apoptosis [29]. Consistently, a higher proportion of 6-TGTP has been shown to correlate with better immunosuppressive efficacy of AZA in CD patients [30]. Therefore, although the overall concentration of 6-TGN is unaltered, NUDT15 heterozygotes, with diminished NUDT15 protein stability, are supposed to have a higher ratio of 6-TGTP at the same dose than the wild genotype, and thus would be more likely to achieve the therapeutic threshold during IBD treatment. This may explain why lower doses of AZA are sufficient to yield favorable clinical outcomes in NUDT15 heterozygotes.

As for the optimal magnitude of the dose reduction in CT genotypes, a balance between both toxicity and efficacy should be considered. Kakuta *et al*. [9] reported that the incidence of thiopurine-induced leukopenia was 39.1% (10/26) in Japanese R139C heterozygotes at a low dose of 0.897 ± 0.303 mg/kg/d; the final maintenance dose was as low as 0.574 ± 0.316 mg/kg/d in this group for safety concerns. However, they did not provide the efficacy data in this context. In the present study, we observed that AZA was effective in remission maintenance in heterozygous IBD patients with an interquartile range of AZA dose <1.0 mg/kg/d [0.83 (0.76–0.95) mg/kg/d]. However, it is noteworthy that the myelotoxicity rate of CT genotype reached up to 45.5% (15/33) at a slightly higher dose of AZA [0.94 (0.79–1.11) mg/kg/d], as mentioned earlier. Combined with these data, it seems plausible that the tolerable and effective doses for a large proportion of East-Asian IBD patients with CT genotype are <1.0 mg/kg/d.

Alternatively, the gradual dose titration also led to a median low dose of AZA [1.05 (0.84–1.35) mg/kg/d] and a relatively low myelotoxicity rate (22.2%) in the wild-genotype patients, which is in accordance with similar studies in East-Asian patients. Yet, the incidence of AZA-induced myelotoxicity reported in Western studies was ≤5% at a standard dose of 2.0–2.5 mg/kg/d [31, 32], implying a potential ethnicity difference even between non-carriers of R139C in East Asia and Western counterparts. Perhaps, this may be caused by other unidentified genetic factors prevalent in East-Asian populations, which requires further investigation. Overall, our results provide preliminary evidence supporting that the final target dose of patients with NUDT15 CT genotype could be set to <1.0 mg/kg/d and that of the CC genotype could be set higher but there is no need for it to be the same as the Western standard dose.

The present study has several limitations that should be discussed. First, the nature of this single-center retrospective design may inevitably include some unknown bias. However, it is due to the single center that the use of AZA is in a relatively consistent manner given that different centers in China have different habits in adjusting to the ultimate doses, as no consensus has been achieved in the final dose in China. Second, we did not test NUDT15 phenotypes (NUDT15 enzyme activity, related metabolite concentration). In fact, new methods have been developed to distinguish 6-TGTP from 6-TGN but they are currently too immature to put into clinical settings [3]. Alternatively, in most parts of China, including our medical center, even the testing of 6-TGN has not been routinely carried out. As a retrospective observational study, metabolite information cannot be provided for analysis. Future prospective studies can be designed to determine whether 6-TGN-guided dose adjustments in NUDT15 wild-genotype patients may be better than the conventional step-up dosing methods in achieving a balance between AZA-related efficacy and toxicity.

In conclusion, patients with CT genotype seem to be more sensitive to AZA in terms of both toxicity and efficacy. Based on the results of our study, we suggest that patients with CT genotype need a smaller step for dose escalation <25 mg/d and might well benefit from a significantly lower target dose of <1.0 mg/kg/d using a step-up dosing strategy to achieve a balance between AZA-related toxicity and efficacy in East-Asian IBD patients.

## Supplementary data

Supplementary data is available at *Gastroenterology Report* online.

## Authors’ contributions

Y.X. and Z.H.R. designed the study; Y.X., H.Y.L., M.Z., C.W.C. and J.S. collected and analysed the data; Y.X. drafted the manuscript; Y.Q.Q. and Z.H.R. revised the manuscript. All authors read and approved the final manuscript.

## Funding

This work was supported by National Natural Science Foundation of China [Grant No. 81670497, 81770545].

## Supplementary Material

goaa021_Supplementary_Data
