# Supplementary figures and images for: NUDT15 genotyping during azathioprine treatment in patients with inflammatory bowel disease: implications for a dose-optimization strategy
Source: Gastroenterol Rep (Oxf). 2020 Jun 26;8(6):437–44. doi: 10.1093/gastro/goaa021 (PMC7793196; doi:10.1093/gastro/goaa021)

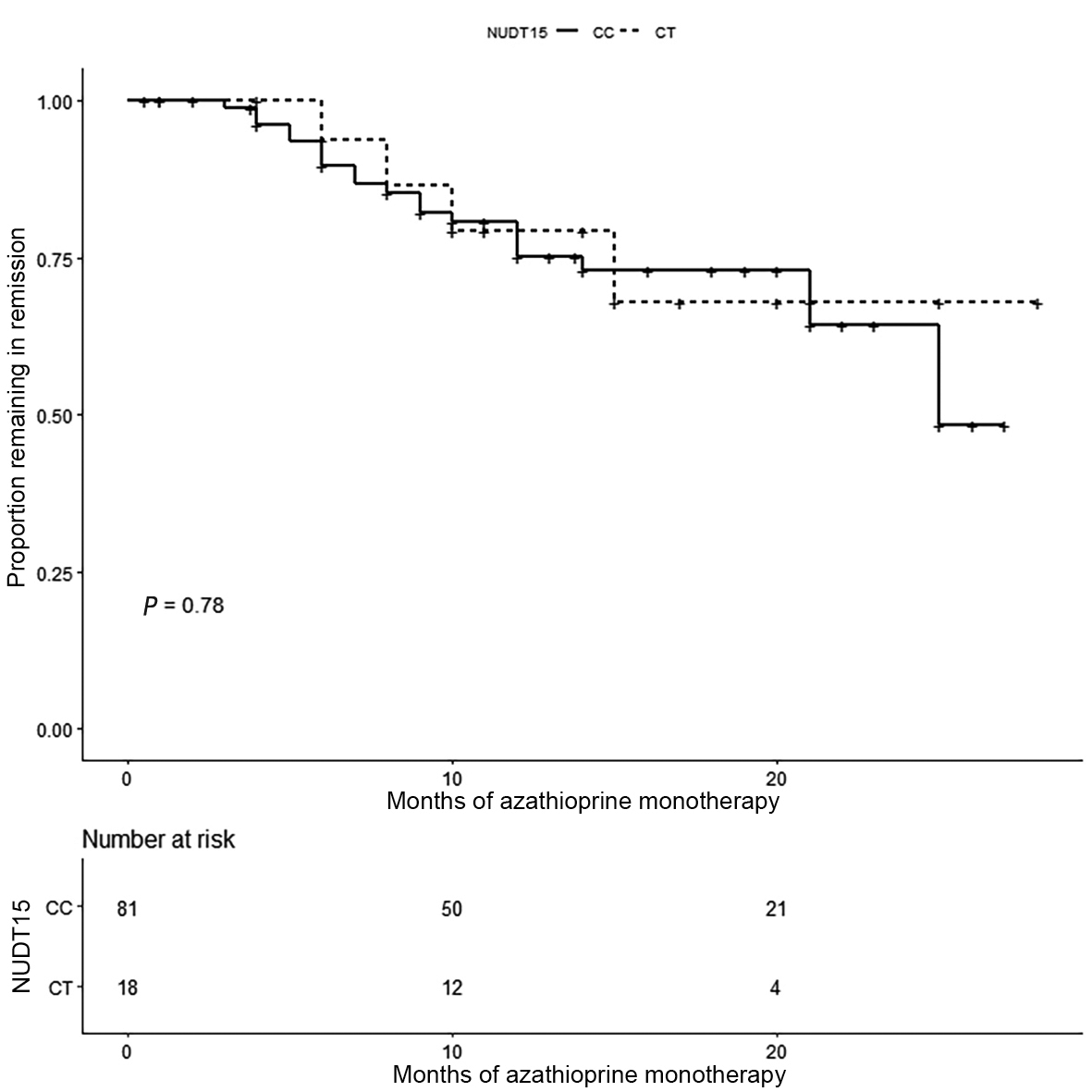

Supplement: goaa021_Supplementary_Data [file goaa021_supplementary_data.png]
